# The Biella paradox: the resilience of plant foraging in a post-industrial pre-alpine area of Northern Italy

**DOI:** 10.1186/s40529-025-00486-8

**Published:** 2025-11-21

**Authors:** Mousaab Alrhmoun, Naji Sulaiman, Sofia Villa, Michele Filippo Fontefrancesco, Andrea Pieroni

**Affiliations:** 1 University of Gastronomic Sciences, Piazza Vittorio Emanuele II 9, 12042 Pollenzo , Italy; 2 Department of Sociology, Università Cattolica del Sacro Cuore, Largo Fra Agostino Gemelli, 1, 20123 Milan, Italy; 3 Department of Medical Analysis, Tishk International University, Erbil, 4001 Iraq

**Keywords:** Cultural heritage, Ethnobotany, Foraging practices, Socio-economic change, Traditional ecological knowledge (TEK)

## Abstract

**Background:**

This study explores the continuity and transformation of wild plant foraging practices in Biella, Piedmont (northwestern Italy), over the past 55 years. The aim was to assess how cultural, economic, and environmental shifts have shaped local ethnobotanical knowledge and practices, using a 1970 survey as a baseline for comparison. Ethnobotanical fieldwork was conducted with 15 local informants to document current wild plant uses for food and herbal teas.

**Results:**

The resulting dataset of 82 species was compared with a historical record of 93 species to identify patterns of continuity, loss, and innovation. Three main patterns emerged: (1) the disappearance of certain traditionally foraged plants, not due to ecological absence but largely because of socio-economic changes like the decline of pastoralism and the loss of daily interaction with mountain environments (2) the emergence of new foraging practices involving species that grow near settlements, linked to evolving lifestyles and land use; and (3) a relatively robust preservation of traditional ecological knowledge when compared to other Alpine areas. This resilience is attributed to the area’s geographic marginality, the socio-economic aftermath of the textile industry’s collapse, and the strength of local traditions such as home gardening and communal land use.

**Conclusion:**

Wild plant foraging remains a living tradition in Biella, marked by both persistence and adaptation. The findings underscore the dynamic nature of ethnobotanical knowledge and its potential role in sustainability, food security, and cultural heritage preservation amid rural and peri-urban change.

## Background

Traditional Ecological Knowledge (TEK) plays a crucial role in the resilience of mountain communities, where human–nature relationships are often shaped by ecological constraints, remoteness, and socio-political marginalization. In such regions, traditional ecological knowledge is not merely a cultural remnant but a living system that adapts to change while anchoring communities to their environments (Alrhmoun et al. 2025a, b; Collantes and Pinilla 2004; Mattalia et al. 2023). In mountain contexts, this knowledge has often been transmitted orally and practiced through necessity, particularly during times of scarcity. Beyond survival, the foraging, preparation, and use of wild plants reveal intricate connections between ecosystems, memory, and community practices (Pieroni and Giusti 2009; Ladio 2017).

However, this knowledge is not static. It evolves or sometimes erodes in response to broader socio-economic transformations such as migration, land use changes, and shifting dietary habits (Sulaiman et al. 2023; 2024). In some cases, marginality itself has paradoxically helped preserve traditional ecological knowledge, as demonstrated in the Sangone Valley of the Italian Alps, where traditional ecological knowledge has endured despite being commodified through tourism (Fontefrancesco and Pieroni 2020). Migration, changes in land use, and shifts in dietary habits have all contributed to the discontinuation or reinvention of plant use (Mattalia et al. 2023). Yet, this erosion is uneven. In some contexts, traditional ecological knowledge is actively maintained or even revitalized, influenced by factors such as community cohesion, local pride, or new forms of valuation like culinary tourism and wellness trends.

Motivations for wild plant gathering also play a role in shaping knowledge continuity. People may forage for food security, medicinal purposes, cultural heritage, or simply for recreational and aesthetic enjoyment. These drivers reflect different levels of knowledge depth and types of engagement with the landscape. In Europe, wild food plants are often gathered by women, and gathering practices are frequently embedded in broader household economies and values.

The Biella province in Northern Italy presents a compelling setting to explore these dynamics. Nestled in the Alps, Biella is known for both its rich natural landscapes and its industrial textile history. The area has experienced significant socio-economic shifts over the past decades, from industrial prosperity to economic restructuring and demographic changes. Despite this, foraging traditions have remained a part of local identity, particularly in mountainous areas of the province (Vinai and Sulis 2017). This dual identity of Biella as both an industrial and mountain territory offers a unique lens for examining the persistence and transformation of plant knowledge (Sella, 1970s).

In the 1970s, a local scholar, Professor Ezio Sella, conducted a detailed ethnobotanical study documenting the wild food plants used in the Biella area. Building on Sella’s work, this study explores the evolution of wild plant knowledge in Biella by comparing past and present data. Through a combination of historical records, ethnobotanical interviews, and participatory research, we aim to understand which plants are still known and used, how preparation and gathering practices have changed, and what these reveal about cultural resilience, innovation, and memory in a rapidly transforming socio-ecological landscape.

## Materials and methods

### Fieldwork and data collection

**Fig. 1:**
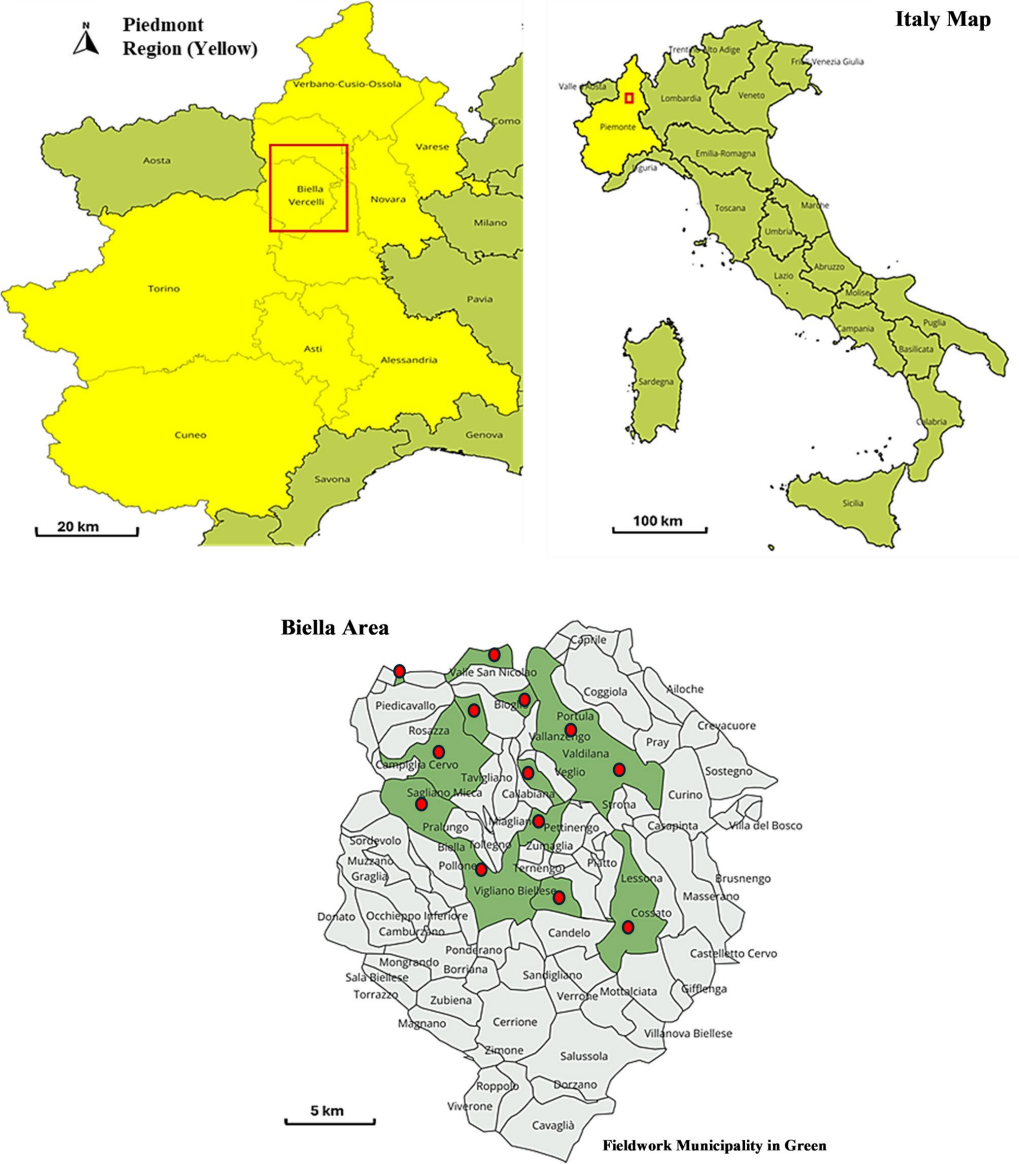
Study area map showing fieldwork sites (in green) and the municipalities of the biellese area, Italy. The first author created this map using QGIS version 3.40.6

This study employs a qualitative ethnobotanical approach to investigate the persistence and transmission of wild plant knowledge in the Biellese area (Piedmont, NW Italy) (Fig. 1). Fieldwork was carried out from December 2024 to February 2025 in many municipalities chosen to reflect the province’s varied ecological and cultural landscapes. These sites span a spectrum from lowland and foothill towns such as Biella, Cossato, and Pettinengo to mid-mountain settlements like Mosso and Camandona, and extend to high-altitude or Walser-rooted villages, including Rassa, Rimella, and Campiglia Cervo (Fig. 1).

This study builds on Sella’s study: *Flora Popolare Biellese*, which documented local wild plants used for culinary and medicinal purposes. The ethnobotanical prospection in the Biella area was conducted in the 1970s, with the results subsequently published by Sella (1992).

We focused specifically on plants with gastronomic significance, reflecting the cultural value of wild flora in Biella. Informants were selected through snowball sampling, starting with those known for their foraging or subsistence knowledge.

A total of 15 participants (11 women and 4 men), ranging from 30 to 85 years old, were interviewed. Thirteen participants were long-term residents of rural or mountainous areas, while two urban dwellers maintained strong familial links to foraging traditions.

Participants exhibited diverse educational backgrounds, from primary to upper secondary levels. Most older informants (born before 1960) recalled periods of rural hardship or post-war subsistence, thus preserving rich, experiential plant knowledge, a profile that included retired farmers, shepherds, and housewives. Meanwhile, a few younger informants (aged 30–50) displayed a revivalist interest in wild plants, frequently motivated by personal hobbies, ecological activism, or affiliations with local botanical and cultural associations.

Data were collected through semi-structured interviews, allowing for an in-depth exploration of informants’ knowledge, experiences, and cultural practices related to wild plant foraging. Interviews were open-ended and encouraged participants to share details about plant names primarily folk names, while scientific names were assigned by the research team based on these folk identifications, along with harvesting practices, preparation methods, and the social contexts in which this knowledge was acquired and transmitted. Particular attention was given to sources of knowledge transmission, including intergenerational learning, local literature, and community-based initiatives. As the study progressed, interviews were complemented by participant observation. Informants were invited to lead foraging walks in their local environments, offering direct insights into plant identification, gathering habits, and ecological awareness in situ. All interviews were conducted in Italian and subsequently transcribed for thematic and comparative analysis.

To ensure taxonomic accuracy, scientific names of wild plants cited in both historical and contemporary sources were verified using authoritative databases such as Flora Europaea, along with standard floristic guides. Plant identification was further supported by specimens previously collected, classified, and deposited in regional herbaria during prior ethnobotanical fieldwork conducted in the Western Alps (Fontefrancesco and Pieroni 2020; Fontefrancesco et al. 2022).

### Data analysis

Thematic analysis was performed to analyze the collected data by focusing on key themes such as the persistence of plant knowledge, generational transmission, and the socio-cultural and ecological factors influencing foraging practices.

The statistical analysis aimed to explore the continuity and change in local herbal knowledge regarding wild plant species in Biella. The analysis compared the presence and use of plants between the 1970 study by Sella and the present study, using descriptive and inferential statistical methods implemented in SAS 9.4 and R version 4.4.3.

Descriptive statistics were calculated to summarize the frequency of species reported in both the 1970 and current studies, as well as the local uses and the corresponding local names. The frequency of plant species presence across the two time periods was summarized using PROC FREQ in SAS 9.4, while the diversity of knowledge was explored using the Shannon Index in R. These indices helped quantify the diversity of plants known and used in the Biellese region, considering the variety of local uses and species richness. A Venn diagram was used to visualize the overlap between the 1970 and current studies, showing the plants that were consistently reported, lost, or newly introduced. The diagram emphasized the species consistently reported in both periods, indicating strong retention of traditional herbal knowledge. This approach highlighted how some species reported by Sella were no longer recognized, while others had been newly added by informants or recent sources.

Hierarchical cluster analysis was used to identify groups of plants that were similarly used across the two study periods. The analysis helped reveal whether any new patterns of plant parts use had emerged or if traditional plant knowledge had remained consistent over time.

Special attention was paid to how the informants’ recollections and current practices related to the plants identified in Sella’s 1970s survey. Comparisons were drawn between past and present plant knowledge, considering the extent to which traditional foraging has been maintained or eroded over the decades.

## Results

### Botanical diversity

**Fig. 2:**
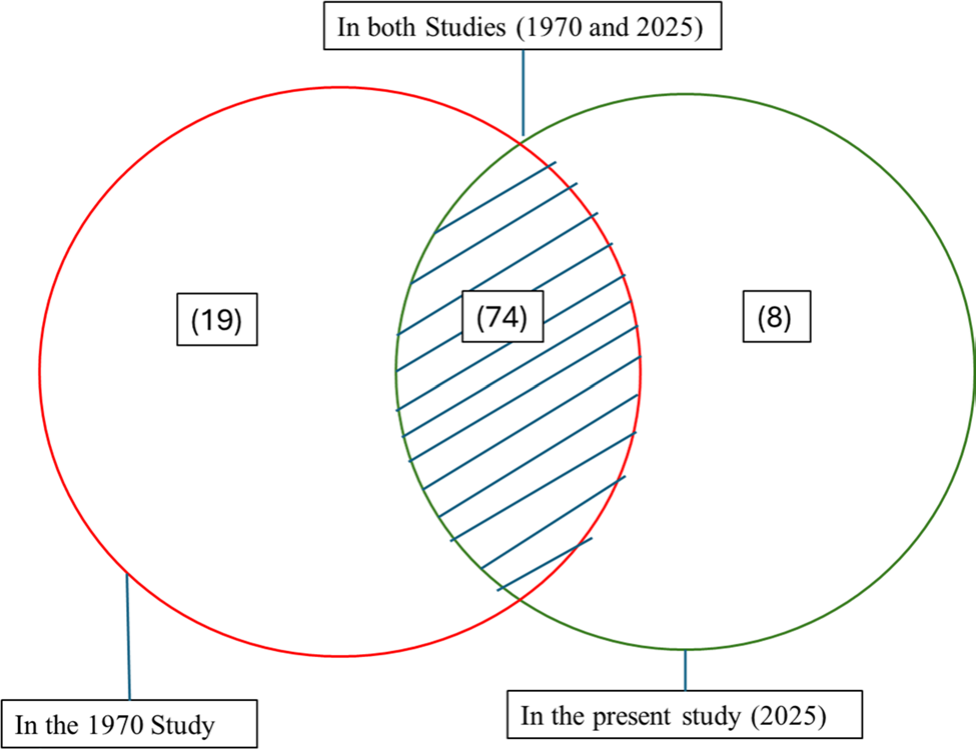
Venn diagram of the recorded species

A total of 39 botanical families were recorded in the study, with the most represented families being Asteraceae, Rosaceae, Polygonaceae, Brassicaceae, Lamiaceae, Boraginaceae, Apiaceae, and Campanulaceae. Across these families, 101 botanical species were documented. Of these, 93 species were already reported in the 1970 study, while 8 species, *Reynoutria japonica* Houtt., *Equisetum arvense* L., *Robinia pseudoacacia* L., *Rosa canina* L., *Salvia pratensis* L., *Tanacetum parthenium*(L.) Sch.Bip., *Trifolium pratense* L., and *Valeriana locusta* L., were newly observed in the present 2025 survey for gastronomic uses (Table 1, Fig. 2).

**Table 1:** Species recorded in “flora popolare biellese” and compared with the data collected in the present study

Scientific Name	Family	1970 Study	Present Study	Local Name	Parts Used	Preparation/Use	Elevation Range (m a.s.l)	Habitat	Ecological categories	Frequency of Citation)(Present study
*Achillea erba-rotta* All.	Asteraceae	Yes	No	Artemisi	Leaves	Liquor	2000–2800	Alpine meadows, rocky slopes, scree on calcareous soils	Mountain/Alpine/ Subalpine	
*Achillea millefolium* L.	Asteraceae	Yes	Yes	Millafó, Müfel	Leaves	Liqueurs; frittatas; mixed herb soups; herbal tea	0–1800	Subalpine grasslands, montane meadows, open forests	Mountain/Alpine/ Subalpine	+
*Alchemilla xanthochlora* Rothm.	Rosaceae	Yes	No	Erba stella	Leaves	Raw or cooked leaves	600–2700	Moist alpine and subalpine woods, shaded forest understory	Mountain/Alpine/ Subalpine	
*Allium ursinum* L.	Amaryllidaceae	Yes	Yes	Ai dal bissi, Ai salvej	Leaves, bulbs, flowers (for decoration)	Pesto for fresh tomatoes	0–1500	Moist deciduous woodlands in lowlands and valleys	Moist/Wetland	+++
*Allium vineale* L.	Amaryllidaceae	Yes	Yes	Ai salvej	Leaves, bulb	Pesto for tomini; chopped like chives	0–1000	Marshy fields, wet meadows, streamside vegetation	Moist/Wetland	+
*Anchusa officinalis* L.	Borraginaceae	Yes	No	Bigulossa	Leaves	-	0–1500	Dry, sunny slopes, roadsides, and disturbed soils	Dry/Calcareous	
*Arnica montana* L.	Asteraceae	Yes	Yes	Arnica	Flowers	Aroma for grappa	500–2500	Heaths and moors in nutrient-poor alpine soils	Mountain/Alpine/Subalpine	++
*Artemisia absinthium* L.	Asteraceae	Yes	Yes	Incens	Leaves	Aroma for grappa	0–1100	Rocky outcrops, screes, stony wastelands (low to mid-elevation)	Dry/Calcareous	++
*Artemisia indica* Willd.	Asteraceae	Yes	Yes	Grisantem salvej	Flowers, leaves	Aroma for grappa (less refined), soups with mixed herbs	0–2000	Road verges, forest edges, scrubland (broad elevation range)	Urban/Ruderal	++
*Aruncus dioicus* (Walter) Fernald	Rosaceae	Yes	Yes	Aspar salvej	Young shoots	Cooked like asparagus, blanched in vinegar, and preserved in oil	200–1800	Mountainous moist forests, rich woodland edges	Mountain/Alpine/ Subalpine	+++
*Bellis perennis* L.	Asteraceae	Yes	Yes	Margarita	Raw or cooked leaves, flowers	Mixed herb soups, raw in salads	0–2000	Urban and rural lawns, grassy roadsides	Urban/Ruderal	+
*Borago officinalis* L.	Borraginaceae	Yes	Yes	Burasu	Leaves	Breaded, fried, soups (Friday steaks)	0–1400	Ruderal areas, fallow fields, disturbed grasslands	Urban/Ruderal	+
*Bunias erucago* L.	Brassicaceae	Yes	No	-	-	-	0–2000	Disturbed areas across elevation zones	Urban/Ruderal	
*Caltha palustris* L.	Ranunculaceae	Yes	No	Armèj	Leaves and buds	Pickled flower buds, cooked	0–2000	Wetlands, fens, moist woodlands	Moist/Wetland	
*Campanula rapunculus* L.	Campanulaceae	Yes	Yes	Rampùn	Leaves, flowers, root	Cooked	0–1500	Dry limestone grasslands, calcareous roadsides	Dry/Calcareous	+
*Campanula trachelium* L.	Campanulaceae	Yes	Yes	Arbëte salvèie	Leaves, flowers, root	Cooked	0–1500	Humus-rich hedgerows and low forests	Moist/Wetland	+
*Cardamine hirsuta* L.	Brassicaceae	Yes	No	Grasùn salvej	Leaves	Salads (raw)	0–1400	Urban cracks, walls, damp disturbed places	Urban/Ruderal	
*Cardamine pratensis* L.	Brassicaceae	Yes	No	Grasùn di pra	Leaves	Salads (pungent flavor)	0–1500	Moist submontane meadows, streambanks	Moist/Wetland	
*Carlina acaulis* L.	Asteraceae	Yes	Yes	Cardùn	Receptacles	Raw or cooked like artichokes	0–2000	Dry calcareous pastures and chalk slopes	Dry/Calcareous	++
*Carum carvi* L.	Apiaceae	Yes	Yes	Sciriel, ciré	Seeds	Flavoring grappa, soups	200–2000	Moist arable lands, montane meadows	Moist/Wetland	++
*Chenopodium album* L.	Amaranthaceae	Yes	No	Farinët	Leaves, sprouts, seeds	Raw or cooked	0–2000	Fields and ruderal roadsides	Urban/Ruderal	
*Chenopodium bonus-henricus* L.	Amaranthaceae	Yes	Yes	Barcùi, spinaci di montagn	Leaves, shoots	Cooked like spinach; gnocchi	500–2000	Productive pastures, farm edges, rural roadsides	Urban/Ruderal	+++
*Cichorium intybus* L.	Asteraceae	Yes	Yes	Sicorja salveja	Leaves	Salads	0–1200	Low-elevation waste land and rocky margins	Urban/Ruderal	+
*Cirsium erisithales* Scop.	Asteraceae	Yes	No	Cardùn, lacèt	Leaves	Cooked in soups	400–1800	Montane woods, stony slopes, stream edges	Mountain/Alpine/ Subalpine	
*Clematis vitalba* L.	Ranunculaceae	Yes	No	Vialbra	Young shoots	Cooked (soups, frittata, risotto)	0–1200	Warm, moist climates on alkaline soils	Moist/Wetland	
*Cornus mas* L.	Cornaceae	Yes	Yes	Curnàl, Curnàlin	Berries	Jam (after pit removal)	0–1400	Well-drained soils from lowland to upland	Dry/Calcareous	+
*Crocus vernus* (L.) Hill	Iridaceae	Yes	Yes	Galët	Flowers	Used in spring salads	0–1500	Submontane grassy habitats and foothills	Mountain/Alpine/ Subalpine	++
*Daucus carota* L.	Apiaceae	Yes	No	Pastana	Leaves, flowers	Leaves cooked; flowers fried in batter	0–1400	Dry fields and ruderal zones	Urban/Ruderal	
*Epilobium montanum* L.	Onagraceae	Yes	Yes	Lacët	Leaves (raw)	Raw leaves in salad with watercress	0–1500	Moist gravel banks, disturbed woodlands	Moist/Wetland	+
*Equisetum arvense* L.	Equisetaceae	No	Yes	Cua d cavàl	Fertile shoots (cooked)	Cooked like asparagus; infusion with sterile stems	0–2000	Damp woods, lowland pastures, stream edges	Moist/Wetland	+++
*Eruca vesicaria* subsp. *sativa* (Mill.) Hegi	Brassicaceae	Yes	No	Rìcula	-	-	0–400	Mediterranean ruderal land, olive groves, tracks	Urban/Ruderal	
*Fagus sylvatica* L.	Fagaceae	Yes	Yes	Fó	Beech nuts	Previously used for oil extraction	0–1500	Moist, humid lowland to upland habitats with well-drained soils	Moist/Wetland	++
*Fragaria vesca* L.	Rosaceae	Yes	Yes	Freia	Fruits	Jam	0–2000	Trails, rural roadsides, hillsides, stone walls (broad range)	Urban/Ruderal	+
*Gentiana acaulis* L.	Genzianaceae	Yes	Yes		Roots	Digestive liquor (infusion)	2000–2700	High alpine pastures, rocky alpine slopes	Mountain/Alpine/ Subalpine	++
*Geum montanum* L.	Rosaceae	Yes	Yes	Fiur dal cucu	Roots, flowers	Digestive liquor (infusion)	500–2500	Subalpine to alpine meadows and rocky terrain	Mountain/Alpine/Subalpine	++
*Helianthus tuberosus* L.	Asteraceae	Yes	Yes	Tupinabò	Tuber	Cultivated and eaten, e.g., with bagna cauda	0–500	Disturbed lowland fields, roadsides	Urban/Ruderal	++
*Humulus lupulus* L.	Cannabaceae	Yes	Yes	Lavartìn, vartis, aspar salvej	Sprouts	Cooked like asparagus, marinated in vinegar (carpione)	0–1200	Riparian woods, moist hedgerows, riverbanks	Moist/Wetland	+++
*Hypochaeris glabra* L. and *Hypochaeris radicata* L.	Asteraceae	Yes	Yes	Patasciùn, patassun	Leaves	Cooked or raw in salads	0–1200	Dry grasslands and sandy lowlands	Dry/Calcareous	+++
*Juglans regia* L.	Juglandaceae	Yes	Yes	Nós	Unripe fruits	Making Nocino liquor	0–1200	Cultivated zones and deciduous forests with fertile soils	Urban/Ruderal	+++
*Juniperus communis* L.	Cupressaceae	Yes	Yes	Snévro	Berries	Liquors, sausages, as an aroma	0–3500	Dry, sandy or rocky soils in open clearings and uplands	Dry/Calcareous	+++
*Knautia arvensis* (L.) Coult.	Caprifoliaceae	Yes	Yes	Spinas salvèj, Scabiosa	Leaves, cooked	In a pan with oil or butter, after blanching them. Served sprinkled with Parmesan	0–2000	Limestone-based meadows, hills, and pastures	Dry/Calcareous	+++
*Lapsana communis* L.	Asteraceae	Yes	Yes	Galinëtte, Galine Grasse	Leaves, raw or cooked	-	0–1400	Disturbed shady places in rural or urban areas	Urban/Ruderal	+
*Laurus nobilis* L.	Lauraceae	Yes	No	Lauro	Leaves	As an aroma	0–400	Coastal Mediterranean zones, warm lowlands	Dry/Calcareous	
*Leucanthemum vulgare* Lam.	Asteraceae	Yes	No	Maragarita müfel	Leaves, cooked	Mainly used as decoration	0–2000	Moist fields, meadows, riparian lake and river edges	Moist/Wetland	
*Lunaria annua* L.	Brassicaceae	Yes	Yes	Midaje del papa	Leaves, cooked or raw; seeds	Cooked like peas	0–400	Urban and rural ruderal areas, waste sites, thickets	Urban/Ruderal	+
*Malva neglecta* Wallr.	Malvaceae	Yes	Yes	Riundèla, malva	Leaves	Soup (risotto around, with a potato as well), laxative. It is said that drunkards used to eat it the day after to recover from the aftereffects. It is dried to make an herbal tea, with a slice of lemon.	0–1800	Anthropogenic grasslands, cultivated fields, mountain slopes	Urban/Ruderal	+++
*Matricaria chamomilla* L.	Asteraceae	Yes	Yes	Camamila	Herbal teas	Infusion	0–1500	Ruderal fields, roadsides, low to montane habitats	Urban/Ruderal	++
*Melissa officinalis* L.	Lamiaceae	Yes	Yes	Melisa	Refreshing infusion	Infusion	0–1000	Gardens, urban edges, lowland roadsides	Urban/Ruderal	++
*Mentha* spp.	Lamiaceae	Yes	Yes	Mènta salveja	Leaves	Refreshing infusion	0–1200	Moist soils in wetlands and shaded sites	Moist/Wetland	+++
*Myosotis scorpioides* L.	Borraginaceae	Yes	Yes	Fior dal bambin	Young shoots	Mixed herbs soups	0–2000	Fens, marshes, and wet field edges to subalpine zones	Moist/Wetland	+
*Nasturtium officinale* R. Br.	Brassicaceae	Yes	Yes	Carsùn, grasùn	Leaves, raw	In salads, a mayonnaise can be made with it	0–2000	Streams, springs, riparian corridors	Moist/Wetland	+++
*Ornithogalum pyrenaicum* L.	Asparagaceae	Yes	Yes	Aspar salvej	Young shoots, immature inflorescences	Cooked like asparagus, boiled and eaten with butter and a sprinkle of Parmesan	0–1500	Woodland margins, hedgerows, and road verges	Moist/Wetland	+++
*Oxalis acetosella* L.	Oxalidaceae	Yes	Yes	Pamblin	Leaves, raw or cooked	In salads	100–2000	Moist shady habitats, montane moors and rocks	Moist/Wetland	+++
*Papaver rhoeas* L.	Papaveraceae	Yes	No	Papavër	-	-	0–2000	Fields, disturbed grounds, roadsides across elevations	Urban/Ruderal	
*Parietaria officinalis* L.	Urticaceae	Yes	Yes	Murajola	Young plants, leaves	In soups, with potatoes, and frittata	0–400	Urban walls, rocky lowland waste places	Urban/Ruderal	+
*Persicaria bistorta* Samp.	Polygonaceae	Yes	Yes	Bargùj, biavëtta	Leaves, cooked or raw; roots	Soup with potatoes and rice, biavëtta e sausissa (salsiccia)	800–2000	Damp grasslands, streambanks in montane zones	Moist/Wetland	+++
*Persicaria maculosa* Gray	Polygonaceae	Yes	Yes	Sabiasc	Leaves	-	0–1200	Wet ditches, lowland wetlands, riparian areas	Moist/Wetland	+
*Phyteuma betonicifolium* Vill.	Campanulaceae	Yes	Yes	Inflorescence	Leaves, immature inflorescence	Cooked like spinach, used in risottos, soups, and paired with cooked salami (erbëtte grasse)	600–2700	Montane to alpine meadows and rocky slopes	Mountain/Alpine/Subalpine	+++
*Phyteuma ovatum* Honck.	Campanulaceae	Yes	Yes	Masuchët, Erbëte	Leaves, inflorescence	Soup with potatoes, carrots, celery, and pieces of lean cheese	800–2000	Alpine meadows and rocky grasslands; prefers alkaline soils	Mountain/Alpine/Subalpine	+++
*Phytolacca americana* L.	Phytolaccaceae	Yes	Yes	Uva dal mèrlo	Known for its toxic saponins	-	0–400	Lowland pastures, woodland edges, wastelands, and clearings	Urban/Ruderal	++
*Pilosella portae* (Willk. ex T.Durand & B.D.Jacks.) Mateo & Greuter	Asteraceae	Yes	Yes	Masciuch ad la Madonna	Leaves	Mixed salad with other herbs	200–2000	Alpine grasslands and rocky slopes with sparse woody plants	Mountain/Alpine/ Subalpine	+
*Pimpinella major* Huds.	Apiaceae	Yes	Yes	Scalëta	Leaves, cooked	Used in mixed salads	0–2200	Burned forests, montane meadows, waysides; calcareous soils	Mountain/Alpine/ Subalpine	++
*Plantago lanceolata* L.	Plantaginaceae	Yes	No	Lengui d’can	Leaves, cooked	Used in mixed herb soups	0–2200	Fields, meadows, lawns, roadsides, woodland edges	Urban/Ruderal	
*Polypodium vulgare* L.	Polypodiaceae	Yes	Yes	-	Rhizome	Chewed raw, tastes like licorice	100–3000	Moist and shaded areas; woodlands, rocky crevices	Moist/Wetland	+
*Portulaca oleracea* L.	Portulaceae	Yes	Yes	Pursclana, Erba Grasa	Leaves	Used in salads and sour soups	0–1500	Fields, gardens, disturbed areas, rural roadsides	Urban/Ruderal	++
*Primula vulgaris* Huds.	Primulaceae	Yes	Yes	Vióla	Leaves, flowers	Used in frittatas and soups	0–2000	Moist woodlands, hedgerows, and upland grasslands	Moist/Wetland	+
*Prunus laurocerasus* L.	Rosaceae	Yes	No	Lauru	Drupes	Infused in alcohol for aroma	0–400	Forest edges, scrublands, ornamental in parks and gardens	Urban/Ruderal	
*Pulmonaria officinalis* L.	Borraginaceae	Yes	Yes	Spinas salvej	Leaves	Cooked	100–1500	Humus-rich soils in shaded woodlands	Moist/Wetland	+ +
*Ranunculus repens* L.	Ranunculaceae	Yes	Yes	Armèi	Leaves	Toxic, sometimes consumed	0–2000	Meadows and fields on rich, damp soils, including gravelly areas	Moist/Wetland	++
*Reynoutria japonica* Houtt.	Polygonaceae	No	Yes	-	-	-	0–1200	Riparian zones, wetlands, ditches, and fencelines	Moist/Wetland	++
*Robinia pseudoacacia* L.	Fabaceae	No	Yes	Gasiia	Flowers	Batter-dipped or candied flowers, used in cakes	0–1000	Forests, disturbed rural and urban areas	Urban/Ruderal	++
*Rosa canina* L.	Rosaceae	No	Yes	Rösa dal cucu	Berries	Rosehip berry jam	0–2000	Hedges, woodland margins, scrublands, and grasslands	Urban/Ruderal	+
*Rubus ulmifolius* Schott	Rosaceae	Yes	Yes	Ruèi	Young shoots	Eaten with mozzarella, in frittatas, or soups	0–1200	Calcareous soils in hedgerows and woodland margins	Dry/Calcareous	+
*Rumex acetosa* L.	Polygonaceae	Yes	Yes	Pancuccu, Pamplüch	Leaves	*Frichj del marghé*; frittatas; soup; flavoured butter	0–2000	Open meadows, roadsides, and grasslands	Urban/Ruderal	+++
*Rumex acetosella* L.	Polygonaceae	Yes	Yes	Erba cucca	Leaves	Frittatas; soup	0–2000	Dry acidic grasslands and heathlands	Dry/Calcareous	+++
*Rumex alpinus* L.	Polygonaceae	Yes	Yes	Lavassa	Central veins of the leaves, cooked	Wild rhubarb jam; *frichj del marghé*	800–2000	Mountain pastures, alpine meadows, and subalpine zones	Mountain/Alpine/ Subalpine	+
*Rumex crispus* L.	Polygonaceae	Yes	Yes	-	-	-	0–1500	Disturbed lowland and upland soils, roadsides, fields	Urban/Ruderal	+
*Rumex obtusifolius* L.	Polygonaceae	Yes	Yes	Lavaza	-	-	0–2000	Moist, nutrient-rich meadows and pastures	Moist/Wetland	+
*Rumex pulcher* L.	Polygonaceae	Yes	No	Côi marìn	-	-	0–1800	Sandy and dry soils in grasslands and roadsides	Dry/Calcareous	
*Rumex scutatus* L.	Polygonaceae	Yes	Yes	Panchicco salvaj	-	-	100–2800	Alpine meadows and rocky high slopes	Mountain/Alpine/ Subalpine	+
*Salvia pratensis* L.	Lamiaceae	No	Yes	Savia salveja	Leaves, cooked	“You can dry sage, make herbal tea with a slice of lemon, rosemary, or fresh sage.”	0–1500	Grasslands, meadows, rural roadsides	Urban/Ruderal	++
*Sambucus nigra* L.	Viburnaceae	Yes	Yes	Sambür	Flowers, fruits	Fried in batter, wine, or syrup with elderflowers; jams with fruit	0–1400	Moist hedgerows, woodland edges, and forest soils	Moist/Wetland	++
*Sanguisorba minor* Scop.	Rosaceae	Yes	Yes	Pimpinela, Scalëtte	Leaves	Aromatic herb for salads, soups, cheese, and vegetables	0–2000	Calcareous grasslands and dry open fields	Dry/Calcareous	+
*Scabiosa columbaria* L.	Caprifoliaceae	Yes	Yes	-	Spring soups: stewed with salame, baked with bread soup	-	0–2000	Calcareous meadows and dry grasslands	Dry/Calcareous	+
*Silene dioica* (L.) Clairv.	Caryophyllaceae	Yes	Yes	-	Young shoots, raw or cooked	-	0–2000	Moist streambanks, woodlands, and hedgerows	Moist/Wetland	+
*Silene flos-cuculi* (L.) Greuter & Burdet	Caryophyllaceae	Yes	Yes	Erba dal marmoti	Leaves, cooked	They are edible and grow near streams.	0–2000	Wetlands, bogs, irrigation channels, and wet meadows	Moist/Wetland	++
*Silene vulgaris* (Moench) Garcke	Caryophyllaceae	Yes	Yes	Varsóla	Leaves and flowers; young shoots, raw or cooked	Frittata, panfried, in pulenta grisa, vegetarian meatballs	0–2000	Disturbed meadows, open woods, and fields	Urban/Ruderal	+++
*Sonchus oleraceus* L.	Asteraceae	Yes	Yes	Lacët	Leaves, basal rosettes, shoots (before flowering), cooked	-	0–1500	Disturbed soils, roadside verges, and waste areas	Urban/Ruderal	+
*Sorbus aucuparia* L.	Rosaceae	Yes	Yes	Jumél	Berries	Grappa, rowanberry, and pear marmalade	0–2000	Mountain woods, rocks, and scrublands at high elevations	Mountain/Alpine/ Subalpine	+
*Symphytum tuberosum* L.	Borraginaceae	Yes	No	Burasu salvej	Young leaves	Mixed herbs soups	0–2000	Semi-shaded riverbanks, woods, and fields	Moist/Wetland	
*Tanacetum parthenium* (L.) Sch.Bip.	Asteraceae	No	Yes	-	Leaves, flowers	Infusion for digestion	0–1000	Mountain scrub, walls, rocky areas; avoids acidic soils	Mountain/Alpine/Subalpine	+
*Tanacetum vulgare* L.	Asteraceae	Yes	Yes	Tanèia	Leaves	Digestive herbal tea, fried leaves, flavoring for salads or frittata, fruit salads or desserts	0–2800	Waste ground, hedgerows across altitudinal gradients	Urban/Ruderal	++
*Taraxacum* sect. *Taraxacum* F.H.Wigg.	Asteraceae	Yes	Yes	Sicória	Every part of the plant	Buds preserved in oil like capers; jam (apples and lemon added)	0–1800	Cultivated fields, gardens, crop edges, rural roadsides	Urban/Ruderal	+++
*Thymus pulegioides* L.	Lamiaceae	Yes	Yes	Pulesc	Raw leaves, dried leaves, cooked leaves	Used as an aroma in dishes	0–2000	Anthropogenic meadows, fields, disturbed lands	Urban/Ruderal	+++
*Tragopogon pratensis* L.	Asteraceae	Yes	Yes	Barbabùch	Leaves, young shoots	Barbabuc in sauce with béchamel and Parmesan; cooked like asparagus; used in savory pies or frittatas	0–2000	Meadows, pastures, dunes, and waste habitats	Urban/Ruderal	+
*Trifolium alpinum* L.	Fabaceae	Yes	Yes	Erba dal büru	Flowers	Risottos, fried in batter, soups	1600–2200	Alpine and subalpine acidic grasslands	Mountain/Alpine/ Subalpine	+++
*Trifolium pratense* L.	Fabaceae	No	Yes	Triföi viulèt	Whole plants	Flower heads fried in batter; sangria can be made with it	0–2000	Riverbanks, meadows, and field margins	Moist/Wetland	+
*Urtica dioica* L.	Urticaceae	Yes	Yes	Urtia	Young shoots	Cooked for preparations like gnocchi, rice, and soups	0–2000	Riverbanks, floodplains, forest edges, and shores	Moist/Wetland	+++
*Vaccinium myrtillus* L.	Ericaceae	Yes	Yes	-	-	-	500–2800	Acidic woodlands, heaths, and moorlands	Dry/Calcareous	++
*Valeriana locusta* L.	Caprifoliaceae	No	Yes	Lacët	Leaves, raw	Salad with hard-boiled egg, pesto, pan-fried with garlic and oil, and in soups with rice	0–1400	Dry soils in cultivated fields, dunes, hedgerows	Dry/Calcareous	+++
*Viola* spp.	Violaceae	Yes	Yes	Viola, Viuleta	Leaves, flowers	Aromatic violet tea, violet grappa; risotto and malastre with Viola tricolor	0–2000	Woodlands, meadows, grasslands; species-dependent habitats	Generalist (could apply to multiple categories depending on species)	+++

Whereas No means the plant is absent from his work. The frequency of citations is categorized as follows: +++Most frequent – widely known and commonly collected. ++Not frequent – known but rarely collected. + Rare – recognized but not gathered. Local names are reported in Piedmontese dialect unless otherwise indicated; a few names are in Italian

**Fig. 3:**
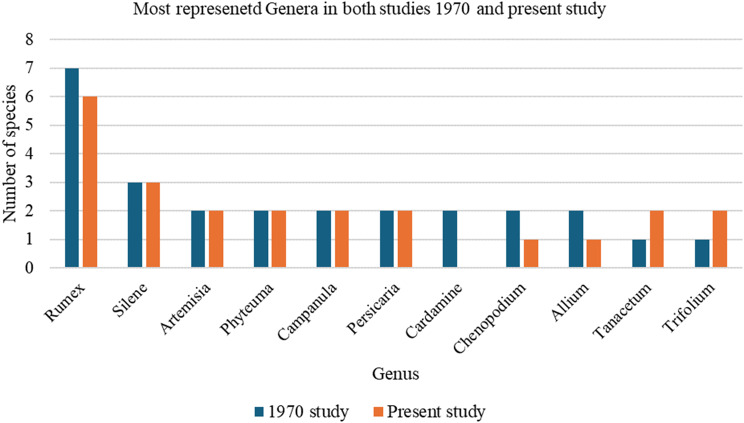
Comparison of the most represented genera in the 1970 and 2025 studies based on the number of species documented

In the two studies, a total of 83 genera were identified. The genus *Rumex* was the most diverse, with seven species. The genera *Silene* and *Hypochaeris* each contained three species, while the following genera each included two species: *Persicaria*, *Artemisia*, *Geum*, *Campanula*, *Achillea*, *Cardamine*, *Allium*, *Phyteuma*, *Chenopodium*, *Tanacetum*, and *Trifolium.* The remaining genera each contained only one species. In a 1970 study, a total of 72 genera were recorded. Among these, the most represented were *Rumex* (7 species), *Silene* (3 species), and *Cardamine, Persicaria, Chenopodium, Artemisia, Phyteuma, Campanula,* and *Allium*, each with 2 species (Fig. 3).

In comparison, the present study, conducted in 2025, documented 67 genera. The most represented genera in this dataset were *Rumex* (6 species), *Silene* (3 species), *Artemisia*, *Phyteuma*, *Tanacetum*, *Campanula*, *Persicaria*, and *Trifolium*, each with 2 species.

A comparative analysis reveals that 66 genera were common to both studies, indicating a substantial overlap in the botanical diversity recorded across the two periods. Notably, the most represented shared genera include *Rumex* (6 species), *Silene* (3 species), and *Artemisia*, *Trifolium*, *Tanacetum*, *Campanula*, *Phyteuma*, and *Persicaria* (each with 2 species) (Fig. 3). Interestingly, eleven genera (*Salvia*, *Equisetum*, *Trifolium*, *Erythronium*, *Rosa*, *Galium*, *Tanacetum*, *Geum*, *Valeriana*, *Peucedanum*, and *Robinia*) were recorded exclusively in the 1970 study. Each was represented by a single species and was noted for uses other than gastronomic purposes, as specified in that study.

**Fig. 4:**
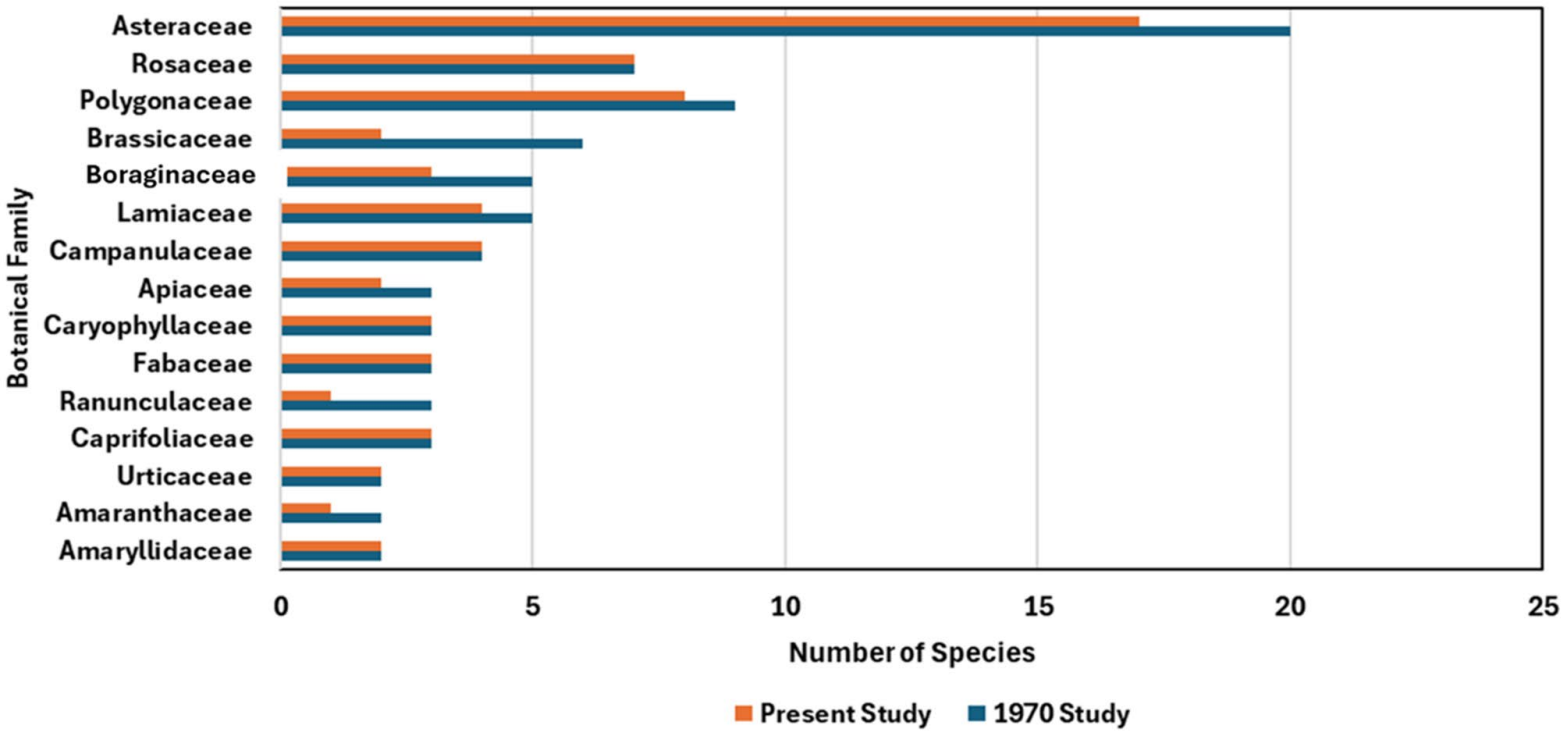
Comparison of the most represented botanical families in the 1970 and 2025 studies *updated*

In the 1970 study, a total of 36 botanical families were recorded, with the most represented being *Asteraceae* (20 species), *Polygonaceae* (9), and *Rosaceae* (7) (Fig. 4). In the present study (2025), 34 families were identified, with the most prominent being *Asteraceae* (17 species), *Polygonaceae* (8), and *Rosaceae* (7). While *Asteraceae*, *Polygonaceae*, and *Rosaceae* remained the most dominant families in both studies, a general decrease in the number of recorded families and species per family was observed in 2025, particularly within *Brassicaceae* and *Rosaceae.*

**Fig. 5:**
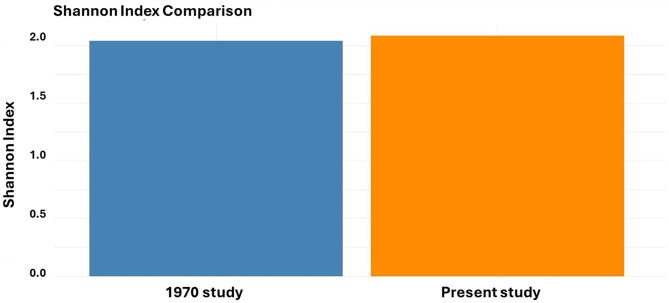
Comparison of the shannon diversity index of foraged plant species between the 1970 study and the present study

The Shannon Index for the 1970 study was 2.04, while the present study yielded a slightly higher value of 2.09 (Fig. 5). This small increase in the Shannon Index suggests a modest rise in the diversity of foraged species over the past 40–50 years. Although the difference is minimal, it indicates that the diversity of wild food plant knowledge has remained relatively stable in the Biella. area.

### The evolution of culinary traditions through time

In the 1970s, most wild plant species in the Biella area were closely tied to local gastronomy. They were used in traditional liqueurs, digestive infusions, and as aromatic herbs in frittatas, mixed soups, and herbal teas. Leaves were eaten raw in salads or cooked in main dishes, while some plants featured in pesto were served with tomatoes or local cheeses like tomini. Some species were used to flavor local spirits like grappa, including less refined types. Wild plants also helped preserve food, with blanched and pickled shoots stored for later use. The cuisine included wild jams and herb-based dishes such as breaded or fried leaves. These traditions reflect a rich gastronomic heritage rooted in necessity and culture. Notably, some plants featured in local recipes like “Friday steaks” (breaded fried herbs), herbal soups with potatoes and rice, or fillings for vegetarian meatballs (Table 1).

In the 2025 data, 82 botanical species continued to be used for gastronomic purposes (Table 1), while 19 species previously employed for food or drink in 1970 were no longer used in such a way today. These species include *Achillea erba-rotta*, *Ajuga reptans*, *Alchemilla xanthochlora*, *Anchusa officinalis*, and *Bunias erucago*, among others (see Table 1 and Fig. 2).

The study reveals both continuity and adaptation in wild plant use. Traditional liqueurs like Nocino and herbal infusions persist, alongside wild plants in pesto, soups, salads, and fried dishes such as battered flowers. Some plants are cooked similarly to spinach or asparagus. Wild plants flavor spirits like grappa and savory dishes. The making of wild jams, especially from rosehips and wild rhubarb, remains important. Unique dishes like “barbabuc” in béchamel sauce, wild herb risottos, and vegetarian meatballs showcase their culinary versatility today. Aromatic species like *Salvia officinalis* and *Rosmarinus officinalis* are used fresh and dried to make flavorful lemon infusions. Wildflowers such as *Viola tricolor* are used for aromatic teas and flavored liqueurs, highlighting the ongoing importance of wild plants in traditional and modern cuisine (Table 1).

**Fig. 6:**
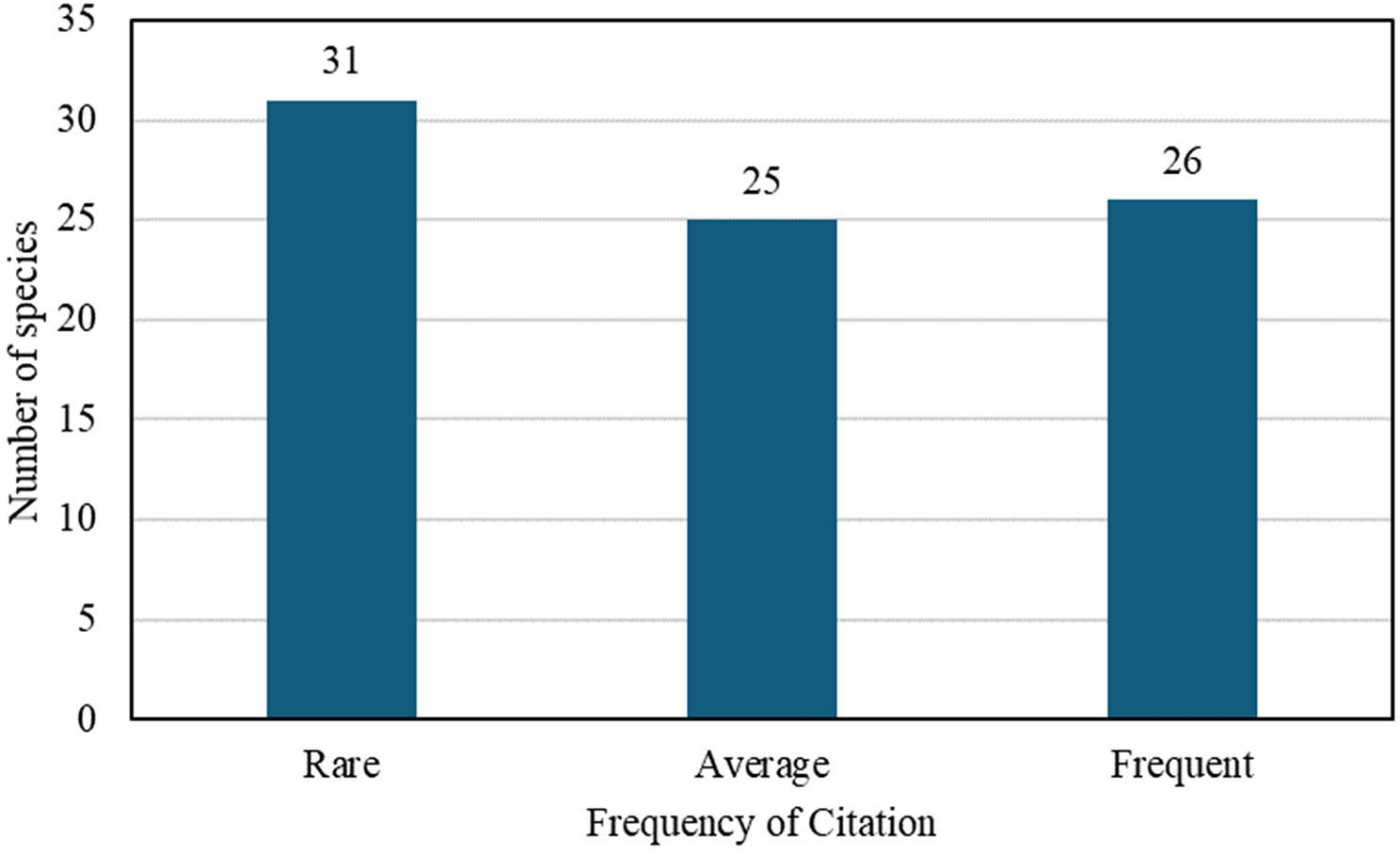
Frequency of citation of plant species in the present study: comparison of rare, average, and frequent species

Although the 1970 dataset lacks citation frequencies, the number of species in each category reveals shifts in plant knowledge. In this study, 31 species were classified as rare, occasionally cited, and potentially fading from local memory, such as *Achillea millefolium*, *Allium vineale*, and *Fragaria vesca*. Twenty-five species were categorized as average, showing moderate recognition and use, including *Arnica montana*, *Artemisia absinthium*, and *Matricaria chamomilla.* Interestingly, *Robinia pseudoacacia*, recorded in the 1970s study, was not encountered in our current survey. Its absence may reflect changes in land use, forestry management, or local vegetation succession, despite the species’ high visibility and invasive potential.(Fig. 6)

Twenty-five species were classified as frequent, showing they remain widely recognized and used locally. Examples include *Allium ursinum*, *Equisetum arvense*, *Juniperus communis*, *Mentha* spp., and *Nasturtium officinale*. For the full list of species, uses, and classifications, see Table 1. Although direct comparisons with the 1970s are unavailable, this distribution suggests that while some knowledge has declined, a strong core of ethnobotanical tradition persists.

### Ecological distribution and elevational trends in wild edible plant species

The data on wild edible plants in the Biella area (Table 2) reveal distinct ecological and elevational trends in species distribution, highlighting the adaptability of these plants to varying environmental conditions. These species were categorized into four main ecological groups: Urban/Ruderal, Moist/Wetland, Dry/Calcareous, and Mountain/Alpine/Subalpine, each of which corresponds to specific elevation ranges and habitat types.

**Table 2:** Ecological categories and botanical species across different elevation zones

Ecological Category	Number of Species	Botanical Species	Elevation (m a.s.l.)
Urban/Ruderal	31	*Artemisia indica*, *Bellis perennis*, *Borago officinalis*, *Bunias erucago*, *Cardamine hirsuta*, *Chenopodium album*, *Chenopodium bonushenricus*, *Cichorium intybus*, *Daucus carota*, *Eruca vesicaria* subsp. *sativa*, *Fragaria vesca*, *Helianthus tuberosus*, *Juglans regia*, *Lapsana communis*, *Lunaria annua*, *Malva neglecta*, *Matricaria chamomilla*, *Melissa officinalis*, *Papaver rhoeas*, *Parietaria officinalis*, *Phytolacca americana*, *Plantago lanceolata*, *Portulaca oleracea*, *Prunus laurocerasus*, *Robinia pseudoacacia*, *Rosa canina*, *Rumex acetosa*, *Rumex crispus*, *Salvia pratensis*, *Silene vulgaris*, *Sonchus oleraceus*, *Tanacetum vulgare*, *Taraxacum sect. Taraxacum*, *Thymus pulegioides*, *Tragopogon pratensis*	0-800
Moist/Wetland	33	*Allium ursinum*, *Allium vineale*, *Caltha palustris*, *Campanula trachelium*, *Cardamine pratensis*, *Carum carvi*, *Clematis vitalba*, *Epilobium montanum*, *Equisetum arvense*, *Fagus sylvatica*, *Geum urbanum*, *Humulus lupulus*, *Leucanthemum vulgare*, *Mentha* spp., *Myosotis scorpioides*, *Nasturtium officinale*, *Ornithogalum pyrenaicum*, *Oxalis acetosella*, *Persicaria bistorta*, *Persicaria maculosa*, *Polypodium vulgare*, *Primula vulgaris*, *Pulmonaria officinalis*, *Ranunculus repens*, *Reynoutria japonica*, *Rumex obtusifolius*, *Sambucus nigra*, *Silene dioica*, *Silene flos-cuculi*, *Symphytum tuberosum*, *Trifolium pratense*, *Urtica dioica*	0-1500
Dry/Calcareous	16	*Anchusa officinalis*, *Artemisia absinthium*, *Campanula rapunculus*, *Carlina acaulis*, *Cornus mas*, *Hypochaeris glabra*, *Hypochaeris radicata*, *Juniperus communis*, *Knautia arvensis*, *Laurus nobilis*, *Rubus ulmifolius*, *Rumex acetosella*, *Rumex pulcher*, *Sanguisorba minor*, *Scabiosa columbaria*, *Vaccinium myrtillus*, *Valeriana locusta*	200-1800
Mountain/Alpine/Subalpine	19	*Achillea erba-rotta*, *Achillea millefolium*, *Alchemilla xanthochlora*, *Arnica montana*, *Aruncus dioicus*, *Cirsium erisithales*, *Crocus vernus*, *Gentiana acaulis*, *Geum montanum*, *Phyteuma betonicifolium*, *Phyteuma ovatum*, *Pilosella portae*, *Pimpinella major*, *Rumex alpinus*, *Rumex scutatus*, *Sorbus aucuparia*, *Tanacetum parthenium*, *Trifolium alpinum*, *Viola* spp.	1000-3000

Thirty-one Plant species from the Urban/Ruderal category, including species such as *Artemisia indica* and *Bellis perennis* (Table 2), are commonly found in lowland areas. These species thrive in the 0–800-meter elevation range, where human activity and land disturbance are more prevalent. However, the presence of these species significantly decreases with elevation.

Thirty-three species recorded in the Moist/Wetland plants, such as *Allium ursinum*, *Caltha palustris*, and *Nasturtium officinale,* are predominantly found in areas with consistent water availability, including wetlands and riparian zones. These species occupy a broader elevational range, from 0 to 1500 meters. Their presence in mid-altitude regions is tied to the abundance of wetland habitats, which are more common in these elevations.

Sixteen Species in the Dry/Calcareous category, such as *Artemisia absinthium*, *Juniperus communis*, and *Vaccinium myrtillus,* are adapted to well-drained, nutrient-poor soils found in calcareous and rocky environments. These species are distributed across a broad range of elevations, from 200 to 1800 meters, and are particularly common in lowland and mid-mountain areas.

In the Mountain/Alpine/Subalpine, nineteen plants were recorded, such as *Achillea millefolium*, *Arnica montana*, and *Gentiana acaulis* (Table 2), which are specially adapted to survive in cold, high-altitude environments. These species are found in the 1000 to 3000 meters elevation range, where the growing season is shorter, and environmental conditions are more extreme.

## Discussion

The present study aims to provide a comprehensive longitudinal comparison of wild edible plant knowledge and use in the Biella area, highlighting both the persistence and evolution of botanical diversity and culinary traditions across five decades. The findings point to a remarkable stability in foraged plant biodiversity, despite modest shifts in species composition and culinary applications, underscoring the resilience of traditional ecological knowledge in this mountain area.

The documentation of 82 botanical species in 2025, nearly the 93 species recorded in 1970, indicates a high degree of continuity in local ecological knowledge. The near-unchanged Shannon Index (2.04 in 1970 and 2.09 in 2025) reinforces this impression of biodiversity stability. This is particularly significant in the context of broader reports of biodiversity loss and erosion of ethnobotanical knowledge across Europe (Łuczaj 2012).

The slight decline in families such as Brassicaceae and Rosaceae could reflect environmental or socio-cultural shifts affecting the availability or desirability of certain taxa. For instance, changes in land management, reduced pasture areas, and abandonment of agricultural terraces in the Alps have been linked to shifts in plant composition (Dibari et al. 2021; Pignatti et al. 2021). Furthermore, the appearance of *Reynoutria japonica*, a known invasive species, aligns with regional ecological concerns, as this plant is rapidly expanding in many parts of Europe, often displacing native flora (Gerber et al. 2008).

Despite some turnover in species used gastronomically, with 11 species lost and 8 gained, the culinary repertoire remains robust. This pattern supports the idea of “selective resilience” in traditional ecological knowledge systems (Berkes et al. 2000), where core knowledge persists while peripheral practices may shift. The sustained use of flagship taxa such as *Rumex* spp. and *Silene vulgaris* suggests cultural attachment to key species embedded in local identity and taste preferences (Pieroni et al. 2002).

Interestingly, several plants not previously used for food (e.g., *Salvia pratensis*, *Viola tricolor*) have gained culinary relevance. This may be influenced by the revival of wild food trends, spurred by gastronomic innovation and local food movements (Menendez-Baceta et al. 2017). The emerging interest in herbal infusions, wild jams, and foraged flowers reflects a blend of tradition and novelty, a phenomenon often described as the “reinvention of tradition” (Johns 1999).

The decline in usage of some traditional plants (e.g., *Chenopodium album*, *Papaver rhoeas*) may reflect a combination of stigmatization of weedy or formerly “poor man’s” foods, changes in taste preferences, or concerns about toxicity and foraging safety, especially among younger generations. On the other hand, the revaluation of certain aromatic and edible wild species now dried, infused, or integrated into fine cuisine echoes trends seen in rural revitalization efforts in Italy and beyond (Redžić 2006; Guarrera and Leporatti 2007).

Overall, the findings support the notion of biocultural resilience, where both biodiversity and cultural practices mutually reinforce each other in sustaining place-based food systems (Barthel et al. 2013).

### Continuity and change in foraging practices in the Biella area

In comparing the current study with that conducted in the 1970s, a relatively modest decline in wild plant foraging is evident. This reduction, although noticeable, is far less dramatic.

A key factor is the geographic marginality of the Biella area, situated on the periphery of Piedmont’s industrial and agricultural hubs. This location has historically insulated the area from the full impact of modernization and intensive agricultural development, helping to preserve traditional rural livelihoods, including the collection and use of wild plants. Another crucial dynamic was the collapse of the textile industry, which had once dominated Biella’s economy. The economic downturn that followed in the latter half of the twentieth century may have contributed to a revitalization of foraging practices, as economically stressed households turned back to land for supplementary resources (Fontefrancesco and Pieroni 2020). Similar trends where economic hardship leads to the revalorization of traditional ecological knowledge have been noted in other parts of Europe (Reyes-García et al. 2013).

Shared traditions with the neighboring Canavese area, where wild food heritage is strong, have supported a local appreciation for foraged plants. Canavese notably became a center of the modern foraging movement in the 1980s, through initiatives such as the Associazione Club Amici Valchiusella, which promoted wild plant knowledge and appreciation. This cultural momentum likely influenced Biella as well, encouraging ongoing engagement with foraging (Fontefrancesco et al. 2023). Local land-use traditions continue to strengthen community ties to nature. Home gardening is still common in Biella, offering both food and ecological awareness. Communal land institutions also play a key role by supporting and regulating sustainable foraging, maintaining it as a culturally and institutionally rooted practice (Pieroni and Giusti 2009). Additionally, the revitalization of traditional food knowledge across Alpine areas of northern Piedmont over the past two decades has influenced wild plant use. In nearby valleys like Valsesia and Ossola, efforts to revive local cultivars and recipes, such as the patata di Otro, reflect a broader cultural shift toward ancestral food practices. Given Biella’s proximity, these trends likely shaped local foraging attitudes, encouraging both the renewed use and culinary reinterpretation of certain species. This highlights the adaptive nature of ethnobotanical practices, shaped by changing cultural contexts.

While the present study focuses primarily on species diversity and usage continuity, detailed culinary knowledge, particularly specific recipes, was only minimally documented. However, understanding how plants are prepared and consumed is crucial to assess whether contemporary traditional ecological knowledge in Biella reflects direct transmission or is instead being reshaped by gastronomic trends, media influences, or cultural revival initiatives, as observed in other post-industrial Alpine communities like Val Sangone (Fontefrancesco and Pieroni 2020). In Biella, anecdotal evidence suggests that traditional preparations using local *Silene vulgaris* (bladder campion) were commonly consumed; *frittata di luppolo selvatico* (hop shoots omelet), and *minestra con ortiche* (nettle soup) remain in use, especially among older generations. Meanwhile, new forms of use, such as wildflower jams or herbal liqueurs, indicate processes of recontextualization, often inspired by gastronomic tourism or media-promoted “wild food” movements.

The persistence of foraging practices in Biella can be considered paradoxical when compared with other post-industrial or industrializing regions, where traditional ecological knowledge has often eroded under the pressure of modernization. For instance, studies in various Alpine valleys of France and Spain (Pardo-de-Santayana et al. 2010) have documented significant declines in plant foraging and knowledge transmission during the second half of the twentieth century, largely attributed to urban migration, agricultural mechanization, and the rise of globalized food systems. Similarly, research in the Balkans has highlighted how industrial development and socio-political transformations often coincided with a weakening of folk plant knowledge (Nedelcheva and Dogan 2015). In contrast, Biella appears to have preserved and, in some respects, revitalized its ethnobotanical heritage despite undergoing intense industrialization. This divergence highlights the resilience of traditional ecological knowledge in Biella and suggests that specific local dynamics such as the strong cultural attachment to the mountainous landscape, the symbolic role of wild plants in regional identity, and the persistence of community-based networks of knowledge exchange may have buffered against the expected erosion. It is precisely this resistance to loss that we frame as the “Biella paradox.”

In sum, the comparison of past and present wild plant use in Biella reveals both continuity and change. While the number of foraged species has slightly declined, the practice remains culturally vital. Foraging traditions persist thanks to a resilient local knowledge system, shaped by geographic marginality, cultural identity, and supportive institutions.

### Limitations and future research

A limitation of this study concerns the legal status of some wild plants that have fallen out of use since 1970. While detailed legislative data is scarce, species like *Caltha palustris*, *Cardamine pratensis*, and *Clematis vitalba* may now be protected or occur in areas where foraging is restricted, such as regional or national parks (Regione Piemonte 2007; Legge 394/1991egione). For instance, *Caltha palustris* grows in protected wetlands, and although not formally safeguarded, species like *Achillea erba-rotta* are of conservation concern in Alpine zones. This study is limited by its reliance on historical ethnobotanical data from a single prior source, which may have underrepresented some uses or taxa. The non-systematic nature of the sampling, along with a limited timeframe, also restricted the diversity of voices and seasonal variation captured.

## Conclusions

The Biella area demonstrates a notable persistence of wild plant foraging practices despite socio-economic transformations and the general decline of ethnobotanical knowledge in Europe. On the side, while ecological and elevational factors may influence the distribution of wild edible plants, the most significant drivers of change in foraging practices are social transformations. Factors such as the decline of pastoralism, reduced time spent in natural environments, and increased urbanization have profoundly affected how plant knowledge is transmitted and practiced. Even when plants remain available in the environment, shifting habits and disconnection from traditional land-based activities mean that many species are no longer sought or used regularly.

This underscores that preserving biocultural heritage requires active efforts to support local traditions, ensure land access, and promote the intergenerational transmission of knowledge. Understanding and addressing these social factors alongside ecological realities is essential for fostering sustainable and adaptive relationships between communities and their natural environments.

## Data Availability

The data that support the findings of this study are presented in the article. Further inquiries should be directed to the corresponding author.
